# Integrated Proteomic and Transcriptomic Analysis of Differential Expression of Chicken Lung Tissue in Response to NDV Infection during Heat Stress

**DOI:** 10.3390/genes9120579

**Published:** 2018-11-27

**Authors:** Perot Saelao, Ying Wang, Ganrea Chanthavixay, Vivian Yu, Rodrigo A. Gallardo, Jack C. M. Dekkers, Susan J. Lamont, Terra Kelly, Huaijun Zhou

**Affiliations:** 1 Integrative Genetics and Genomics Graduate Group, University of California, Davis, CA 95616, USA; psaelao@ucdavis.edu (P.S.); kchantha@ucdavis.edu (G.C.); 2 Genomics to Improve Poultry Innovation Lab, University of California, Davis, CA 95616, USA; ucywang@ucdavis.edu (Y.W.); trkelly@ucdavis.edu (T.K.); 3 Department of Animal Science, University of California, Davis, CA 95616, USA; vsyu@ucdavis.edu; 4 School of Veterinary Medicine, University of California, Davis, CA 95616, USA; ragallardo@ucdavis.edu; 5 Department of Animal Science, Iowa State University, Ames, IA 50011, USA; jdekkers@iastate.edu (J.C.M.D.); sjlamont@iastate.edu (S.J.L.)

**Keywords:** chicken, Newcastle disease virus, RNA-seq, proteomics

## Abstract

Newcastle disease virus (NDV) is a devastating worldwide poultry pathogen with major implications for global food security. In this study, two highly inbred and genetically distinct chicken lines, Fayoumis and Leghorns, were exposed to a lentogenic strain of NDV, while under the effects of heat stress, in order to understand the genetic mechanisms of resistance during high ambient temperatures. Fayoumis, which are relatively more resistant to pathogens than Leghorns, had larger numbers of differentially expressed genes (DEGs) during the early stages of infection when compared to Leghorns and subsequently down-regulated their immune response at the latter stages to return to homeostasis. Leghorns had very few DEGs across all observed time points, with the majority of DEGs involved with metabolic and glucose-related functions. Proteomic analysis corroborates findings made within Leghorns, while also identifying interesting candidate genes missed by expression profiling. Poor correlation between changes observed in the proteomic and transcriptomic datasets highlights the potential importance of integrative approaches to understand the mechanisms of disease response. Overall, this study provides novel insights into global protein and expression profiles of these two genetic lines, and provides potential genetic targets involved with NDV resistance during heat stress in poultry.

## 1. Introduction

Newcastle disease virus (NDV) has become one of the most important avian diseases impacting poultry production and trade worldwide. NDV is a highly contagious viral disease, capable of spreading rapidly among populations of birds and decimating poultry flocks [1]. The World Organization for Animal Health includes virulent forms of NDV as a specific hazard due to the impact that NDV outbreaks have on international trade and animal welfare [2]. Proper vaccination, along with effective use of biosecurity and biosafety measures on poultry farms, have been largely successful in preventing infections by NDV. However, understanding host genetic diversity and heterogeneity in the immune response can aid in supporting these measures and inform upon the genetic factors involved in effectively responding to infection. 

Changing climate conditions is an additional factor that may impact the host’s resistance and response to disease. Extreme weather events have been found to create more frequent [3] and intense [4] disease outbreaks. Studies on seasonality, and the effects of temperature on Newcastle disease outbreaks have noted higher incidences during the hot and dry, and hot and humid seasons [5,6]. Additionally, investigations into the specific effects of heat stress on the immune response in chickens have shown immunosuppressing effects through reduced lymphoid organ weights [7,8], lower circulating antibodies [9], and altered systemic humoral response [10]. 

Several studies have investigated the role that host genetics plays in explaining differences in response to NDV without heat treatment [11,12,13] in chickens. These studies found Fayoumi chickens, native to Egypt and imported to the U.S. in 1954, to be relatively more resistant to the La Sota strain of NDV, with significantly lower viral load and higher circulating anti-NDV antibodies, than Leghorn chickens [12,14]. Furthermore, Fayoumis has demonstrated higher resistance to other viral pathogens such as avian influenza [15] and Marek’s disease [16]. Transcriptome profiling of the two genetic lines during NDV infection has identified many candidate genes and signaling pathways potentially regulating the tissue-specific response to NDV infection [11,12,13]. Specifically, the mucosal surface of the airways in chickens is one of the main sites targeted for infection and entry by respiratory pathogens such as NDV. The innate and adaptive immune response of tissues in the respiratory tract, including the lungs, are of critical importance to limiting the systemic spread of the virus in the bird. 

Large-scale analysis of the proteome can accurately determine the relative abundance of proteins within tissues. Proteomics offers the opportunity to comprehensively investigate the changes in signaling pathways and biological processes that would otherwise be missed by transcriptome sequencing. In addition, quantitative proteomics and the integrative analysis of the transcriptome can provide corroborative evidence for high priority targets to aid in the discovery of unique mechanisms. The objective of this study was to identify candidate genes and proteins involved with the chicken response to NDV infection during heat stress using an integration of proteomic and transcriptomic approaches. We believe this integrated approach will identify novel biomarkers and provide insight into the differences in response to NDV infection during high ambient temperatures between Fayoumi and Leghorn chicken lines.

## 2. Materials and Methods

### 2.1. Experimental Populations and Design

The experimental design of this study has been previously described by Wang et. al. 2018 [17]. Briefly, Fayoumi (M15.2) and Leghorn (GHs 6) inbred chicken lines from Iowa State University (Ames, IA, USA) were used in this study. On the day of hatching, 56 Fayoumi and 55 Leghorn chicks were transported from Iowa State University to the University of California, Davis. Upon arrival, the chicks were housed in temperature- and humidity-controlled chambers in a biosafety level 2 animal facility. Twenty-five individuals from each genetic line were randomly selected and housed in a separate chamber to be used as the control group. From day 1 to day 13 both groups were reared at 29.4 °C and 60% humidity. At 14 days of age, the experimental group was exposed to 38 °C for 4 h, then decreased to 35 °C and maintained at this temperature until conclusion of the trial. The control group was maintained at 29.4 °C and gradually decreased to 25 °C. On day 21, the heat-treated birds were inoculated with 200 μL 10^7^ EID_50_ of the La Sota strain of NDV through both ocular and nasal passages. The control group was mock inoculated with 200 μL of 1X phosphate-buffered saline (PBS). At 2, 6, and 10 days post-infection (dpi), four birds per treatment group per genetic line were randomly selected and euthanized with CO_2_. Lung tissue was harvested and quickly placed into RNA*later* (ThermoFisher, Waltham, MA USA Cat#AM7024) and kept at −80 °C. Additional lung tissue samples were collected at 2 and 6 dpi and snap frozen in liquid nitrogen and then stored at −80 °C until protein extraction. Protein extraction, protein assay, and the in-solution trypsin digestion were performed as reported by Kultz et. al. [18]. The experiment’s procedures were performed according to the guidelines approved by the Institutional Animal Care and Use Committee at the University of California, Davis (IACUC #17853). 

### 2.2. RNA Isolation and Library Construction

Total RNA was extracted from the lung of four individuals per treatment and genetic line for each of the three time points. The lung tissue was homogenized in ice cold TRIzol (ThermoFisher, Waltham, MA USA Cat#15596026) and processed using a standard phenol:chloroform method and precipitated in 100% ethanol. The RNA pellet was then dissolved into water and treated with DNase I (ThermoFisher, Cat#EN0521). Strand-specific RNA library preparation was prepared using the NEBNext Ultra Directional RNA Library Prep Kit for Illumina (NEB, Ipswich, MA USA Cat#E7420S) according to the manufacturer’s instructions. Library validation and quantification was conducted using the Agilent Bioanalyzer High Sensitivity Kit (Agilent, Santa Clara, CA USA Cat#5067-4626) and Qubit dsDNA HS Assay kit (ThermoFisher, Cat#Q32854). The 100 base pair, paired-end sequencing was performed on the Illumina HiSeq2500 system with a minimum sequencing depth of 30 million reads per sample. Sequence data have been submitted through the Sequence Read Archive under accession number: SRP155740.

### 2.3. Protein Identification and Quantification

Protein identification and quantification was performed as previously described by Kultz et. al. (2016) [19]. Briefly, peptides from each sample were separated by reverse phase liquid chromatography and then analyzed by an ImpactHD UHR-QTOF mass spectrometer (Bruker Daltonics, Bremen, Germany). Peak lists were imported into PEAKS 7.5 (BSI, Waterloo, Canada) and PEAKS, Mascot 2.2 (Matrix Science, London, UK) and X!Tandem Cycle [19] search engines used to identify proteins from the MS/MS spectra. Search results from all three methods were combined in Scaffold 4.4 (Proteome Software Inc., Portland, OR, USA) with peptide false discovery rate (FDR) < 0.1, protein FDR < 0.1, and peptides per protein ≥ 2. Label-free quantitative profiling of peptide intensities was performed in PEAKS 7.5, and all identification and profiling data were made publicly available at the CAMP proteome at the University of California, Davis (Davis, CA USA, AC# CAMPDDA00051). Pathway analysis of differentially abundant proteins, DAPs, identified at each timepoint between conditions was done using STRING [20]. The correlation between the log_2_ change in protein abundance and log_2_ fold change in gene expression was calculated on 585 protein-transcript pairs. Spearman’s correlation coefficient and significance value was determined using R’s cor.test function [21] for all timepoints. 

### 2.4. Data Analysis

Four major factors were included for analysis: condition (treated, non-treated), line (Leghorn, Fayoumi), sex (male, female), and time point (2, 6, and 10 dpi). Data at each time point consisted of 16 individuals, four per treatment and genetic line. Raw reads from RNA-seq were trimmed using FastQC [22] to remove duplicates, reads with base quality scores <30, and adapter contamination. These reads were then aligned using STAR [23] to the galGal5 reference genome and Ensembl annotation using default settings. Gene counts were calculated using HTSeq [24] and differential gene analysis was done using edgeR [25]. The statistical model design included the effects of line, condition, sex, and time point. In addition, in order to identify genes that were differentially expressed between genetic lines in response to treatment and, therefore, potentially associated with disease resistance to NDV, the interaction between condition and genetic line was included. Genes were identified as differentially expressed if they had an FDR < 0.05, and an average transcript count >10. Pathway analysis using the DEGs of within line contrasts was performed using Qiagen’s Ingenuity Pathway Analysis software [26]. Z-score cutoff of |z| > 1 identified significantly up- or down-regulated pathways [26]. 

## 3. Results

### 3.1. Differential Gene Expression from within-Line Comparisons

Differential gene expression analysis between treated and non-treated individuals was performed within both Fayoumi and Leghorn chickens at 2, 6, and 10 dpi to explore changes in gene expression induced by NDV infection and heat stress. Comparisons between treated and non-treated Fayoumi chickens at 2 dpi identified 122 differentially expressed genes (DEGs) that were up-regulated and 58 DEGs that were down-regulated (Table 1). The number of DEGs subsequently decreased to 26 up-regulated and 27 down-regulated DEGs at the 6 dpi timepoint. The largest number of DEGs identified within line was at 10 dpi with 173 up-regulated and 358 down-regulated DEGs. For Leghorns, 35 DEGs were up-regulated while only two DEGs were down-regulated at 2 dpi. Principal component analysis to identify sample clustering for the transcriptomic and proteomic data can be found in the Supplementary Figures (Figures S1 and S2). For both the transcriptomic (Figure S1) and proteomic (Figure S2) datasets, the samples appeared to cluster by genetic line and then by dpi. In both datasets, the samples did not appear to cluster by treatment state across all three time points. Very few genes were differentially expressed at 6 dpi with only six DEGs up-regulated and one DEG that was down-regulated. At 10 dpi, Leghorns had only 10 and five DEGs up-regulated and down-regulated, respectively. 

### 3.2. Pathway Analysis of Differentially Expressed Genes 

**Figure 1:**
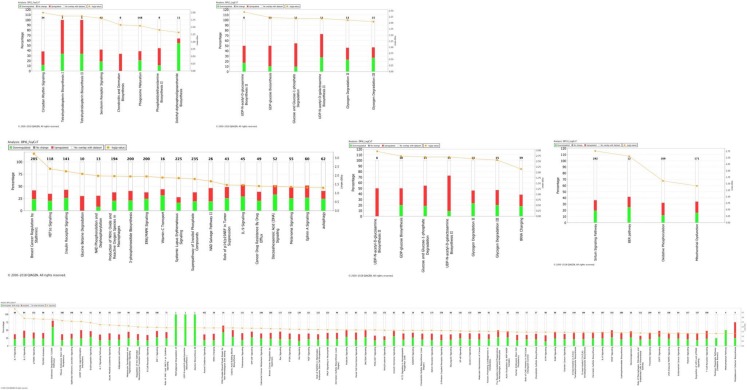
Significantly enriched pathways identified through the Ingenuity Pathway Analysis among the differentially expressed genes by timepoint and genetic line. (**A**) Fayoumi at 2 days post-infection (dpi), (**B**) Leghorn at 2 dpi, (**C**) Fayoumi at 6 dpi, (**D**) Leghorn at 6 dpi, (**E**) Fayoumi at 10 dpi, and (**F**) Leghorn at 10 dpi. The number of genes in each pathway is shown in black, up-regulated (red) and down-regulated genes (green) within the pathway, and the significance value calculated as the −log(*p*-value) is depicted as an orange line.

Ingenuity pathway analysis (IPA) of the DEGs identified in the Fayoumi and Leghorn within line comparisons revealed canonical pathways unique to each genetic line (Figure 1A–F). Fayoumi had eight and 19 significant pathways enriched at 2 and 6 dpi, respectively. Notable pathways include the phagosome maturation (2 dpi), serotonin receptor signaling (2 dpi), IL-9 signaling (6 dpi), ERK/MAPK timepoint (6 dpi), and autophagy signaling (6 dpi) pathways. Pathway enrichment at 10 dpi identified 47 significantly enriched pathways, with 28 of these pathways with a predicted negative Z-score suggesting down-regulation and one pathway with a predicted positive Z-score (Table 2). These down-regulated pathways include IL-8 signaling, B cell receptor signaling, and NF-kappaB signaling which are prominent pathways involved in the immune response. Very few pathways were enriched among the Leghorn DEGs across all three timepoints. At 2 dpi, only six pathways were significantly enriched, and of these pathways the majority were involved with glycogen metabolism including glycogen degradation II, glycogen degradation III, and glucose and glucose-1-phosphate degradation pathways. Similarly, these three glycogen and glucose degradation pathways were again among the seven enriched pathways at the 6 dpi timepoint for Leghorns. Analysis at the 10 dpi timepoint identified only four significantly enriched pathways. 

### 3.3. Quantification of Protein Levels and Differential Protein Abundance 

**Figure 2:**
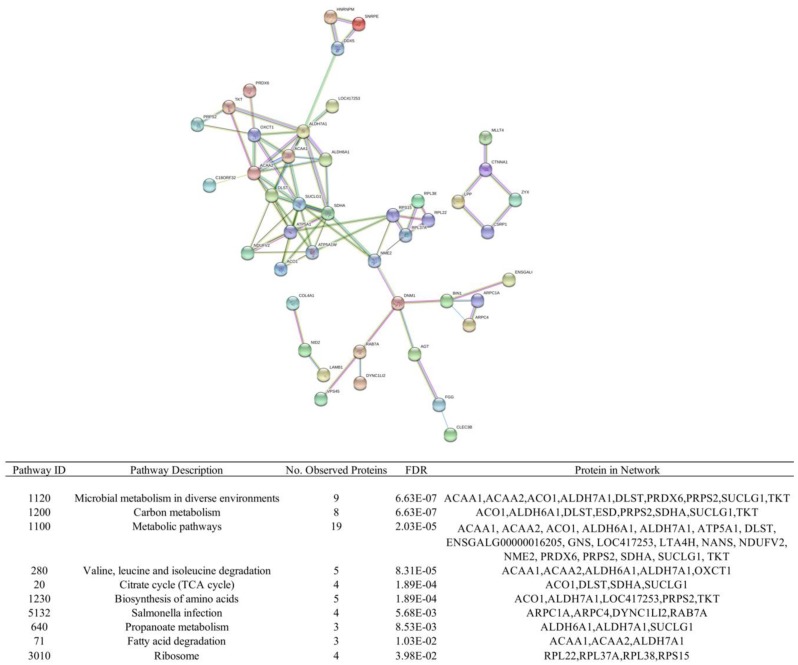
STRING network analysis on DAPs between treated and non-treated Leghorns at 6 dpi. Nodes depict protein-protein interactions. Figure table lists the significantly enriched pathways and their corresponding differentially abundant protein within these pathways.

Proteins from lung tissue were extracted and quantified from the same birds used for transcriptome analysis at 2 and 6 dpi to identify significantly over or under abundant proteins expressed during the early immune response. The primary goal for protein quantification was to investigate the early stages of infection, and the early proteomic response observed within these timepoints. The 10 dpi timepoint was not investigated for protein quantification due to sample limitations and central focus of the early immune response. Comparing treated to non-treated Fayoumis, we found six and 13 DAPs at 2 and 6 dpi, respectively (Table 1). Differentially abundant proteins are proteins that have been identified as differentially abundant in either the treated or non-treated groups. The same comparisons made within Leghorns revealed 10 DAPs at 2 dpi. The largest number of DAPs was identified at 6 dpi with 99 DAPs found to be differentially abundant between treated and non-treated birds. No significant pathways were identified within Fayoumis at either timepoint and for Leghorn at 2 dpi using STRING. However, KEGG pathway enrichment analysis found 10 significant pathways enriched among the 99 DAPs at 6 dpi in Leghorns (Figure 2). Similar metabolic-related pathways identified from the differential gene expression analysis in Leghorns were found among the pathways enriched in the protein dataset. 

### 3.4. Correlation of Change in Protein Abundance and Differential Gene Expression

**Figure 3:**
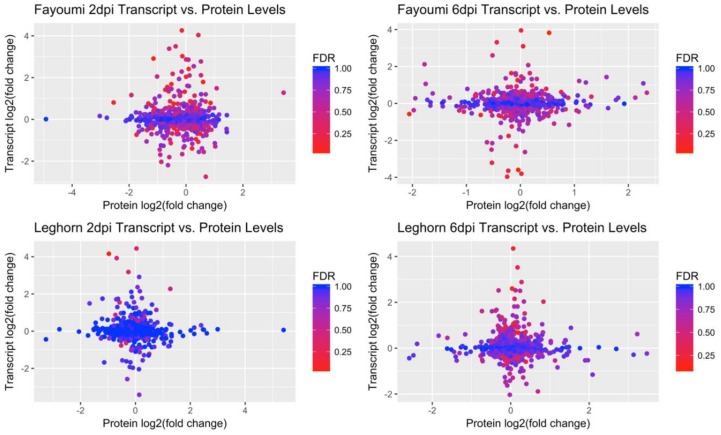
Correlation between the log_2_(fold change) in gene expression and the log_2_(fold change) in protein abundance between treated and non-treated birds at each timepoint. Intensity of each point represents the q-score of the gene.

The strength of the relationship between the change in protein abundance and gene expression changes due to NDV infection and heat treatment were determined for each line and comparison (Table 3). A total of 585 protein-transcript pairs were identified across 2 and 6 dpi in order to observe protein and gene expression correlations (Figure 3). Correlation between the change in protein abundance and gene expression differences due to treatment at both time points in Leghorns and at 2 dpi in Fayoumis were weakly negatively correlated by Pearson correlation analysis. Fayoumi at 6 dpi had weakly positive correlation, however, none of the correlations at any timepoint within either genetic line was significant. Additional comparisons between the normalized read counts and relative protein abundances also failed to find significant association between the two datasets across all comparisons except for non-treated samples at 6 dpi in the Fayoumi line (Additional File 1). 

## 4. Discussion

To our knowledge, this study is the first to simultaneously address the molecular events that occur during NDV infection and heat stress using global proteomic and transcriptomic profiling techniques in chickens. Our study investigated the dynamic protein and gene expression changes induced in the lung by NDV at 2, 6, and 10 dpi to understand the host response during heat stress. Differential gene expression analysis between disease states found interesting differences in response patterns when comparing the relatively resistant Fayoumis to the more disease susceptible Leghorns. In the Fayoumi comparisons, there was a large number of DEGs identified at 2 dpi with the majority of the DEGs (122) significantly up-regulated. Notable immune-related genes include *IL17REL*, which is involved in cytokine-mediated signaling, and *NOX4*, *PRDX1*, *RAB7B*, which are part of the phagosome maturation pathway. Interestingly, very few genes were differentially expressed at 6 dpi, however, results from our group show a similar result in the Harderian gland tissue of the same individuals [14]. The 10 dpi timepoints had many DEGs identified but most of the genes were significantly down-regulated (358). This could be an indication of a resolution of the immune response with genes involved with immunity being down-regulated for the maintenance of tissue homeostasis in Fayoumies. Similar studies in chicken Harderian gland, lung, and trachea have also observed a down-regulation of DEGs at 10 dpi providing further indication that the Fayoumis may have resolved the infection and are returning to homeostasis [11,12,14]. Compared to Fayoumis, Leghorns had very few DEGs identified across all three timepoints in this study. The low number of DEGs may suggest that the lungs of Leghorn birds are not responding as effectively to NDV infection during heat stress. One gene that is of particular interest is *PGM3*, which was significantly up-regulated at both the 2 and 6 dpi timepoints in Leghorns. *PGM3* encodes for a protein that mediates glycogen formation and utilization [27]. Studies on *PGM3* mutants demonstrate immune deficiency [28], decreased cytokine production [29], and increased susceptibility to avian influenza [30]. 

Pathways enriched among the DEGs identified provide insight into the potential mechanisms and gene groups utilized in response to NDV infection during heat stress. At 2 dpi, Fayoumi had significant enrichment in two immune-related pathways, serotonin receptor signaling and phagosome maturation. Serotonin receptor signaling is of particular interest due to its role in hormonal regulation of immune function and high expression amongst immune cells with several immunoregulatory functions such as cytokine secretion and T-cell activation [31]. Differentially expressed genes from 6 dpi had a similar trend in enrichment of immune-related pathways (IL-9 signaling, ERK/MAPK, autophagy signaling) amongst the small number of significant pathways. These pathways may be critically important for the enhanced disease resistance observed in Fayoumis. In addition, IPA predicted down-regulation of enriched pathways at 10 dpi. However, the neuroinflammation signaling pathway was weakly activated at the 10 dpi timepoint within Fayoumis. Velogenic and mesogenic strains of NDV often localize within the central nervous system (CNS) where it can result in systemic disease and rapid mortality [32]. Neuroinflammation can drive the activation of many pro-apoptotic pathways in an attempt to alleviate infection but may also result in increased pathology of neuro diseases [32]. While the activation of this pathway was limited (z-score = 0.45), it is worth noting the potential benefits of the neuroinflammation response observed in the putatively NDV resistant Fayoumis. Fayoumis’ down-regulation and modulation of the immune system to return to homeostasic levels can be viewed as characteristic of an efficient immune response at the early stage of infection. 

While Fayoumis appears to be activating pathways involved with the immune and antiviral response at 2 and 6 dpi, Leghorns were primarily enriched for metabolic-related pathways such as glycogen degradation II and III, and glucose and glucose-1-phosphate degradation at these timepoints. Leghorns appear to be responding to the effects of infection-induced hyperglycemia at both the 2 and 6 dpi timepoints. During infection, it is common to observe increased glucose utilization during increased periods of fasting. Failure of peripheral glucose clearance can lead to hyperglycemia and result in the dysregulation of the immune response [33]. The induction of a fasting state due to both infection and heat stress, and a dysregulation of the glucose-related processes in Leghorn, could potentially explain the poor performance of their immune response. 

Despite the similarities in pathways and genes enriched in the proteome and transcriptome data, significant correlation was not observed between the two datasets. Many studies have shown evidence that protein and mRNA levels tend to have poor correlation, especially amongst diverse cell types [34,35]. This may be due to various post-transcriptional or post-translational events that would be difficult to observe within our datasets. In addition, the in vivo dynamics in our dataset may not necessarily recapitulate the in vitro systems typically reported when comparing the two methodologies. However, our study identified unique DAPs between treated and non-treated groups that were not identified in the transcriptome analysis. Of the DAPs identified in Fayoumi, *ATOX1*, *SFTPA1* and *LL* are of interest due to their prominent roles in the innate immune system and defense against respiratory pathogens. Yeast two-hybrid screens found *ATOX1* to interact with partners in the TRIM family of proteins related to the immune response [36] and inhibition of viral growth [37]. LL and SFTPA1 are surfactant-related proteins predominantly found within the lung and are involved in the defense against respiratory pathogen and modulation of ligand C-type lectin-related-b (Clr-b) proteins [38]. A study in cell lines and mice observed that decreases in surfactant proteins leading to a decrease in clr-b, increase natural killer (NK) cells’ ability to sense virus-infected cells and increase NK cell response [39]. However, within Leghorns, proteomic analysis appeared to capture similar observations of increased metabolic and glucose-related processes seen within RNA expression data. COL1A2 was a protein differentially abundant at 2 dpi between Leghorn treatment groups and family collagen-related genes have been observed to be differentially expressed in studies of NDV infection in the lung and trachea [11,12]. At the 6 dpi timepoint, there was significant enrichment of KEGG pathways which include: metabolic, carbon metabolism, citrate cycle (TCA cycle), and fatty acid degradation. It appears that Leghorns were having a more profound alteration of lung metabolism due to infection that may be causing a dysregulation of their immune response. In vivo studies have observed the magnitude of infection and inflammation which result in profound activation of the glucoregulatory systems [40]. The infection status within Leghorns may be substantial enough to shift the body to a state of metabolic inflexibility, typically observed during high levels of inflammatory stress. 

In summary, comparisons within Fayoumis and Leghorns using protein and gene expression profiling identified unique significant pathways and candidate genes involved in the lung’s response to NDV infection during heat stress. The integration of these two methods allowed for global profiling of the birds’ responses and provided corroborative evidence for observations witnessed in the gene expression and protein studies. Additionally, our study identified potentially important sets of genes and proteins which would have been missed through the independent use of either method. The potential roles and underlying molecular mechanisms involved in NDV response during heat treatment require further investigation into the candidate genes and signaling pathways identified.

## Figures and Tables

**Table 1:** Number of differentially expressed genes (DEGs) and differentially abundant proteins (DAPs) in Fayoumis and Leghorns at each timepoint with a false discovery rate (FDR) < 0.05.

		*Differentially Expressed Genes*		*Differentially Abundant Proteins*	
*Line*	Days Post-Infection	Up-Regulated	Down-Regulated	Up-Regulated	Down-Regulated
*Fayoumi*	2	122	58	0	6
*Leghorn*	2	35	2	5	5
*Fayoumi*	6	26	27	4	9
*Leghorn*	6	6	1	62	37
*Fayoumi*	10	173	358		
*Leghorn*	10	10	5		

**Table 2:** Top canonical pathways significantly enriched between treated and non-treated Fayoumis at 10 dpi. Activated pathways are Z-score > 0 and inhibited pathways are Z-score < 0. NA designates pathways without a predicted activity score.

Canonical Pathway	Z-Score
ERK/MAPK Signaling	−2.24
Thrombin Signaling	−2.24
mTOR Signaling	−2.00
Fcy Receptor-mediated Phagocytosis in Macrophages and Monocytes	−2.00
p70S6K Signaling	−1.89
B Cell Receptor Signaling	−1.89
Colorectal Cancer Metastasis Signaling	−1.89
Mouse Embryonic Stem Cell Pluripotency	−1.63
Rac Signaling	−1.34
Ga12/13 Signaling	−1.34
IL-8 Signaling	−1.34
NF-kappaB Signaling	−1.34
Regulation of eIF4 and p70S6K Signaling	−1.34
NGF Signaling	−1.34
Telomerase Signaling	−1.34
Acute Myeloid Leukemia Signaling	−1.34
Fc Epsilon RI Signaling	−1.00
fMLP Signaling in Neutrophils	−1.00
ErbB Signaling	−1.00
Growth Hormone Signaling	−1.00
EIF2 Signaling	−1.00
FLT3 Signaling in Hematopoietic Progenitor Cells	−1.00
Melanocyte Development and Pigmentation Signaling	−1.00
Renal Cell Carcinoma Signaling	−1.00
Neuregulin Signaling	−1.00
Insulin Receptor Signaling	−1.00
GM-CSF Signaling	−1.00
Sirtuin Signaling Pathway	−0.45
Prolactin Signaling	0.00
PTEN Signaling	0.00
Neuroinflammation Signaling Pathway	0.45
AMPK Signaling	NA
G Beta Gamma Signaling	NA
FcyRIIB Signaling in B Lymphocytes	NA
Renin-Angiotensin Signaling	NA
Role of NFAT in Cardiac Hypertrophy	NA
eNOS Signaling	NA
Huntington’s Disease Signaling	NA
GP6 Signaling Pathway	NA
ErbB4 Signaling	NA
Lymphotoxin B Receptor Signaling	NA
PAK Signaling	NA
CD40 Signaling	NA
Production of Nitric Oxide and Reactive Oxygen Species in Macrophages	NA
alpha-Adrenergic Signaling	NA
P2Y Purigenic Receptor Signaling Pathway	NA
Amyotrophic Lateral Sclerosis Signaling	NA

**Table 3:** Correlation coefficients and *p*-value statistics calculated between the log_2_(fold change) in gene expression and the log_2_(fold change) in protein abundance between treated and non-treated birds at each timepoint.

*Line*	*Days Post-Infection*	*r*	*p*-Value
*Fayoumi*	2	−0.0072	0.86
*Leghorn*	2	−0.053	0.22
*Fayoumi*	6	0.051	0.22
*Leghorn*	6	−0.018	0.65

## Supplementary Materials

The following are available online at http://www.mdpi.com/2073-4425/9/12/579/s1, Figure S1: Principle component analysis of sample clustering of all RNA-Seq samples. Samples appear to cluster primarily due to the genetic line rather than treatment state, Figure S2: Principal component analysis demonstrating the clustering of proteomic samples at A) 2 dpi and B) 6 dpi.
