# Supplementary material for: Integrated Proteomic and Transcriptomic Analysis of Differential Expression of Chicken Lung Tissue in Response to NDV Infection during Heat Stress
Source: Genes (Basel). 2018 Nov 27;9(12):579. doi: 10.3390/genes9120579 (PMC6316021; doi:10.3390/genes9120579)
Supplement: Supplementary file 1 [file genes-09-00579-s001.pdf]

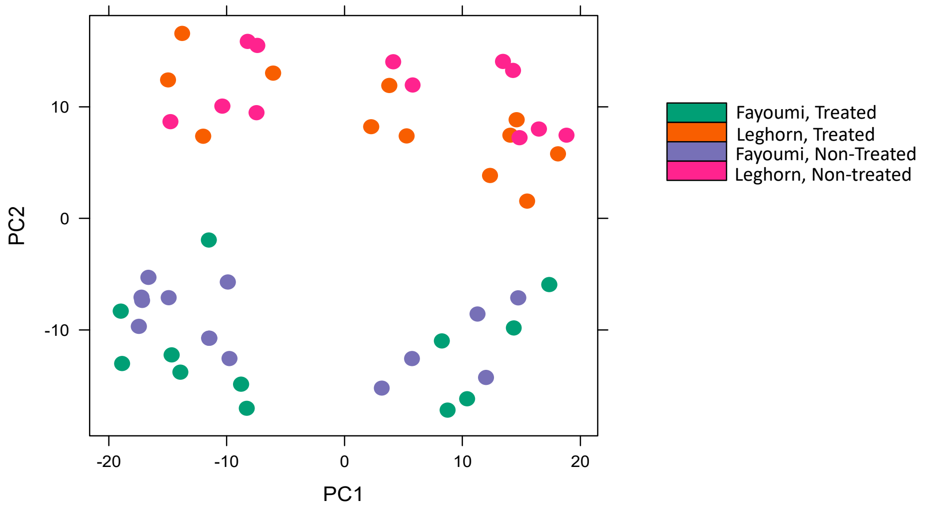

Figure S1: Principle component analysis of sample clustering of all RNA-Seq samples. Samples appear to cluster primarily due to the genetic line rather than treatment state.

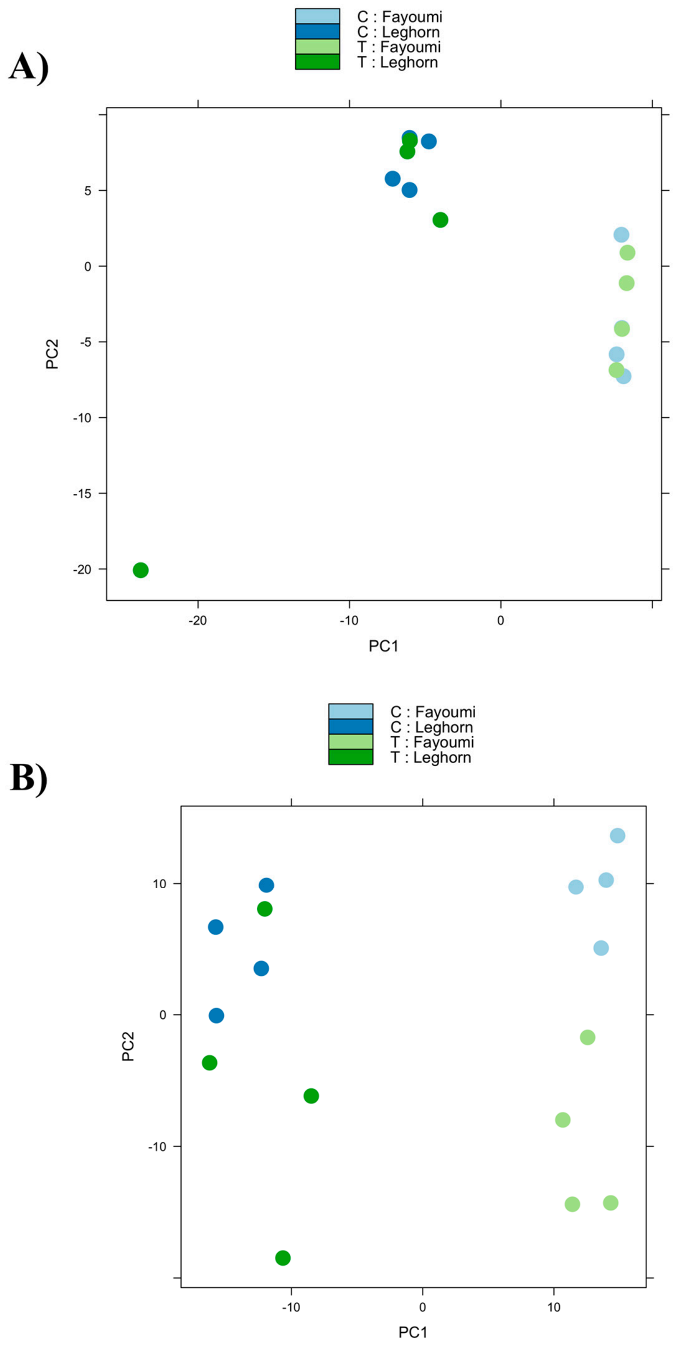

Figure S2: Principal component analysis demonstrating the clustering of proteomic samples at **A)** 2 dpi and **B)** 6 dpi.
